# Effect of genetic deletion and pharmacological antagonism of P2X7 receptors in a mouse animal model of migraine

**DOI:** 10.1186/1129-2377-15-24

**Published:** 2014-05-01

**Authors:** Flóra Gölöncsér, Beáta Sperlágh

**Affiliations:** 1 Laboratory of Molecular Pharmacology, Institute of Experimental Medicine, Hungarian Academy of Sciences, H-1083, Budapest, Szigony u., 43, Hungary; 2 János Szentágothai School of Neurosciences, Semmelweis University School of Ph.D Studies, Budapest, Hungary

**Keywords:** P2X7 receptor, Brilliant blue G, nitroglycerin, mouse model, migraine

## Abstract

**Background:**

Purine receptors participate in peripheral and central sensitization and are associated with migraine headache. We investigated the role of P2X7 receptor (P2X7) in a nitroglycerin (NTG)-induced mouse model of migraine.

**Methods:**

Intraperitoneal NTG injection (15 mg/kg) triggered thermal hyperalgesia in the hindpaws of wild-type C57BL/6J mice, followed by the induction of c-fos in upper cervical spinal cord and trigeminal nucleus caudalis. The effect of genetic deletion of P2X7 and the selective P2X7 antagonist Brilliant Blue G (BBG) were examined on hyperalgesia and c-fos induction.

**Results:**

NTG decreased the paw withdrawal threshold in both wild-type and P2X7 knockout mice. Nevertheless, subacute BBG treatment (50 mg/kg/day i.p.) completely prevented the effect of NTG in wild-type, but not in knockout mice. Whereas P2X7 deficiency differentially affected the expression of c-fos, the average number of fos-immuno-reactive neurons in trigeminal nucleus caudalis, but not in upper cervical spinal cord was lower in BBG-treated wild-type mice after NTG treatment.

**Conclusions:**

Our results show that P2X7 receptors might participate in the pathogenesis of migraine, although upregulation of other P2X receptors probably compensate for the loss of its action in knockout mice. The data also suggest the therapeutic potential of P2X7 antagonists for the treatment of migraine.

## Background

Headache is a common pain disorder affecting an estimated 11% of the general population [1] and the global prevalence of chronic migraine is 0.5-1% [2]. Despite increasing knowledge regarding migraine pathophysiology, drug therapies used for prevention and treatment of symptoms remain unsatisfactory for many patients. Therefore, it is necessary to continue development of new drugs and to identify promising new cellular or molecular targets [3].

Migraine is a complex brain disorder, characterized by predominantly unilateral pulsating head pain, which can be aggravated by routine physical activity and accompanied by neurological symptoms including nausea, vomiting, hypersensitivity to light, sound and smell and visual disturbances as well as cognitive, emotional and motor disturbances. Before and during migraine attacks, headache is accompanied by increased skin sensitivity to touch, heat and cold (cutaneous allodynia) [4,5] and normally innocuous stimuli can be unpleasant and painful.

Among the variety of experimental models of migraine attacks, nitroglycerin (NTG) is amongst the most widely used and accepted approaches, in both animals and human [6]. Several reports show that the clinical use of NTG induces delayed spontaneous headache in migraine patients [7-9] coupled with accompanying symptoms. NTG-derived nitric oxide (NO) activates brainstem regions and neuronal populations which are involved in environmentally triggered migraine attacks [10] and prophylactic agents can block NTG-induced headaches in human [11,12]. Furthermore, NTG can provoke cutaneous allodynia in migraine sufferers [13] including in non-cranial regions [4,5]. Previous studies showed that systemic administration of NTG induces hyperalgesia of the hindpaws, which is inhibited by the antimigraine drug sumatriptan in mice [14]. These observations validate extrafacial thermal and mechanical hyperalgesia as a potential behavioural correlate of migraine in mice subjected to NTG. NTG administration also activates neuronal populations in selected areas of the brain that are primarily involved in the transmission of cephalic pain, including the trigeminal nucleus caudalis (TNC) in rodents [14-16]. Chemical activation of the C-fibers of the trigeminal nerve - leading to transmission of nociceptive information into the brainstem - induces c-fos expression within specific areas of the TNC. This is inhibited by anti-migraine therapeutics, such as olcegepant [17]. These findings indicate that those drugs, which influence c-fos expression in the TNC might have relevance in the mediation of potential anti-migraine effects.

The purinergic signalling system consists of enzymes, transporters and receptors responsible for the action of extracellular nucleotides, particularly ATP and adenosine. The actions of extracellular nucleotides are mediated by various subtypes of ionotropic P2X (P2X1-7) and metabotropic P2Y (P2Y_1_, P2Y_2_, P2Y_4_, P2Y_6_, P2Y_11_, P2Y_12_, P2Y_13_, P2Y_14_) receptors. The potential involvement of the purinergic signalling system in the pathophysiology of migraine was recognized more than thirty years ago [18]. Since then, the role of different subtypes of P2 receptors has been elucidated in different pain modalities, amongst which, the role of P2X3, and various P2Y receptor subtypes have already been proposed and examined in cellular and animal models of migraine [19-24]. ATP is released during spreading depression (SD), a phenomenon thought to underlie aura both in vitro [25] and in vivo [26], which raises the possibility that endogenous purines could act as potential triggers or mediators of migraine.

P2X7 receptors (P2X7) belong to the P2X family of receptors, which are ligand-gated, non-selective cation channels. P2X7 is widely expressed in various cell types involved in pain transmission, including neurons, microglia, satellite glial cells, astrocytes in dorsal root ganglia, trigeminal ganglia and the dorsal horn of the spinal cord e.g. [27-29]. Several studies have demonstrated that P2X7 is involved in the modulation of pathological nociception. P2X7 knockout mice showed a lack of hypersensitivity to mechanical and thermal stimuli [30,31] and P2X7-specific antagonists have consistently been shown to be protective in animal models of inflammatory [32-34] and neuropathic pain [33,35]. Moreover, recent studies have highlighted that variation within the gene encoding P2X7 can affect chronic pain sensitivity in both mouse and human [36]. The role of P2X7, however, has not examined in any whole animal model of migraine.

The objective of this study, therefore, was to examine how genetic deletion and pharmacological blockade of P2X7 affects NTG-induced thermal hypersensitivity and c-fos induction in migraine related CNS areas in mice. We report here that although genetic deletion of P2X7 (P2X7-/-) results in non-significant changes in the nociceptive threshold after NTG treatment, the selective P2X7 antagonist, BBG completely prevents the effect of NTG in wild-type, but not in P2X7-/- mice and also alleviates NTG-induced c-fos expression.

## Methods

### Animals

All experiments were conducted in accordance with the principles and procedures outlined in Guide for the Care and Use of Laboratory Animals of the National Institute of Health. The local Animal Care Committee of the Board of the Institute of Experimental Medicine, Hungarian Academy of Sciences approved all experimental procedures (Permission No: 22.1/3671/003/2008). This study used drug and test naïve, 3-month old (approx. 30 g), male wild-type (P2X7+/+), and P2X7 knockout mice (P2X7-/-). Breeding pairs of P2X7-/- homozygote knockout mice (C57BL/6J based) were originally supplied by Christopher Gabel (Pfizer Inc., Groton, CT, USA). The animals contained the DNA constructs P2X7-F1 (5_-CGGCGTGCGTTTTGACATCCT-3_) and P2X7-R2 (5_-AGGGCCCTGCGGTTCTC-3_), which have previously been shown to produce genetic deletion of P2X7 [37]. Homozygous knockout mice (P2X7-/-) and P2X7+/+ (C57BL/6) littermates were bred in the local animal house (IEM HAS, SPF Unit). The genotype of the mice was tested by PCR analysis as previously described [37]. Mice were housed under standard laboratory conditions with food and water available ad libitum, in a 12-h light–dark cycle at a temperature of 21–23°C. All efforts were made to minimize animal suffering and reduce the number of animals used. Experiments were carried out between 9:00 and 14:00 in the same room in which the animals were housed, whilst perfusion took place in a different room.

### Drugs and treatments

A modified version of NTG model described by Bates et al. [14] was used. Animals received intraperitoneal (i.p.) injections of 15 mg/kg NTG (Nitro POHL®, G. Pohl-Boskamp GmbH & Co. KG, Hohenlockstedt, Germany) or vehicle (49 mg glucose monohydrate/ml) after the measurement of the baseline thermal nociceptive threshold. The dose of NTG was chosen based on literature data [14] and preliminary tests. Sumatriptan succinate (Sigma-Aldrich, Hungary), dissolved in saline was used to validate our experiments: each animal was given i.p. sumatriptan at the dose of 600 μg/kg [14] or saline 5 minutes after NTG administration. The P2X7 antagonist, Brilliant Blue G (BBG, Sigma-Aldrich, Hungary) or its vehicle (0.9% saline), were applied in an identical way, or using two different prophylactic application protocols: acutely (50 mg/kg), it was applied i.p. 30 minutes prior to NTG treatment and after the measurement of the baseline thermal nociceptive threshold on the day of testing. In subacute treatment, mice were treated for 5 consecutive days with the daily doses of BBG (50 mg/kg i.p.) or saline and 30 min after the last injection were subjected to NTG. Selection of dose was based on previous studies [38,39]. All drug solutions were freshly prepared on the day of use.

### Increased temperature hot plate test (ITHT)

Mice were randomly assigned to experimental groups of 10–13 and housed 5 per cage for 1 week prior to experimentation. An Excel protocol was used to randomize the animals, and codes unblinded only after the experiment. Responsiveness of mice to nociceptive stimulation was measured by an increasing-temperature hot plate system (IITC Life Science, Woodland Hills, CA, USA). On the day of testing, animals were habituated to the testing apparatus for 10 min prior to determination of baseline nociceptive threshold. The animals were placed on an electrically heated metal plate that was kept at a constant temperature of 30°C (starting temperature). After the habituation period the plate was heated from the starting temperature with a constant rate of 6°C/min, until the animals showed nocifensive behaviour (frequent pawlifting and/or pawlicking in both front and back paws, jumping).

Heating was then instantly stopped, the animal removed from the apparatus and the plate rapidly cooled. The temperature at which the animal showed the first sign of nocifensive behaviour was taken as the paw withdrawal threshold (PWT), expressed in °C. Approximately 1 hour later, the measurement was repeated and the average of two values was taken as the baseline thermal nociceptive threshold. After the second measurement, the animals received treatment with drugs as described above and one and two hours after nitroglycerin administration, post-drug nociceptive threshold was measured.

### Immunohistochemistry (IHC)

Among animals undergoing behavioural testing, 5–8 mice/experimental group were selected by randomization prior to experimentation and were assigned to be the subject of subsequent perfusion and immunohistochemistry. Two hours following i.p. injection of NTG (15 mg/kg) or vehicle, mice were anaesthetized with ketamine and xylazine (i.p.) and perfused with fixative solution (containing 0.5% borax (Na_2_B_4_O_7_ 10 H_2_O) and 5% paraformaldehyde). Whole brain and spinal cord were removed and fixed for 3 h in 5% paraformaldehyde, and transferred to 30% sucrose in fixative solution for 20–24 h. Samples were then frozen and transverse sections cut at 30 μm. Every fourth section was collected for free-floating IHC. After blocking the endogenous peroxidase activity with 0.3% hydrogen-peroxide, sections were incubated in 0.3% Triton X-100 and 2% normal goat serum for 1 h, followed by incubation with anti**-**fos (raised in rabbit, 1:10000, 72 h at 4°C, sc-52, Santa Cruz Biotechnology). Sections were then washed in PBS before incubation for 1 h in biotinylated goat anti-rabbit secondary antibody, and incubated with avidin-biotin-complex (Vectastain – ABC kit PK-6100 Elite, 1 h). Peroxidase activity was detected with 3,3′-diaminobenzidine (Sigma-Aldrich, Hungary). Mounted and coverslipped sections were microphotographed and fos-immuno-reactive nuclei were counted within both sides of the cervical spinal cord and the TNC using ImageJ software. We counted 30 hemi-sections from each hemisphere per animal (TNC) and 10 hemi-sections from each hemisphere per animal (cervical spinal cord) and calculated the average number of c-fos positive nuclei in each section.

### Statistics

All data were expressed as means ± SEM of *n* observations. For statistical comparison of thermal sensitivity after treatments in P2X7+/+ and P2X7-/- mice, a repeated measures analysis of variance ANOVA was used to identify genotype, treatment and time effects. The number of c-fos positive nuclei in spinal cord and TNC was compared by two-way ANOVA. Where the variance was not homogeneous based on a significant Levene’s test, we used the Kruskal-Wallis nonparametric test. The Fischer LSD test was used for post hoc comparison. For pairwise comparisons, the Student *t* test was used. All tests were performed by the STATISTICA software package (StatSoft, Tulsa, OK, USA).

## Results

### Effect of genetic deletion of P2X7 on NTG-induced thermal hypersensitivity

**Figure 1:**
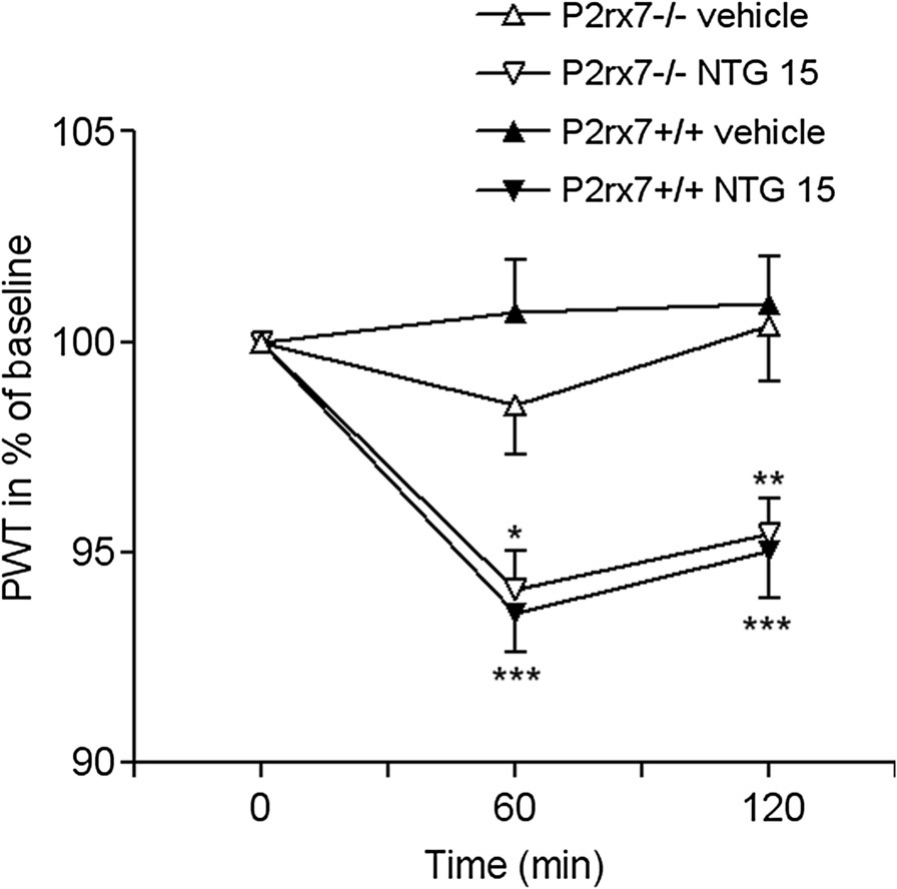
**NTG induces thermal hypersensitivity in wild-type and P2X7 knockout mice.** The changes in nociceptive threshold after i.p. NTG treatment are presented on the graph, as PWT, expressed in % of baseline. PWT was decreased in animals that received 15 mg/kg NTG compared with animals that received vehicle (ANOVA, effect of treatment, F_1,41_ = 37.34, p < 0.0001). NTG-induced decrease in PWT was not significantly different in wild-type and knockout mice. Asterisks denote significant changes between respective PWT values of vehicle- and NTG-treated mice (n = 10-13 animals/group; ANOVA + Fischer LSD post hoc test, *P < 0.05, **P < 0.01, ***P < 0.001).

The baseline nociceptive threshold in wild-type (P2X7+/+) mice was 45.95 ± 0.14°C (n = 68). NTG significantly and time-dependently reduced PWT, when compared with vehicle treatment (at 1 h: 100.70 ± 1.25%, n = 12; and 93.56 ± 0.95%, n = 13; 2 h: 100.92 ± 1.13%, n = 12; and 95.06 ± 1.13%, n = 13, in vehicle and NTG-treated mice, respectively, ***P < 0.0001; Figure 1).

**Figure 2:**
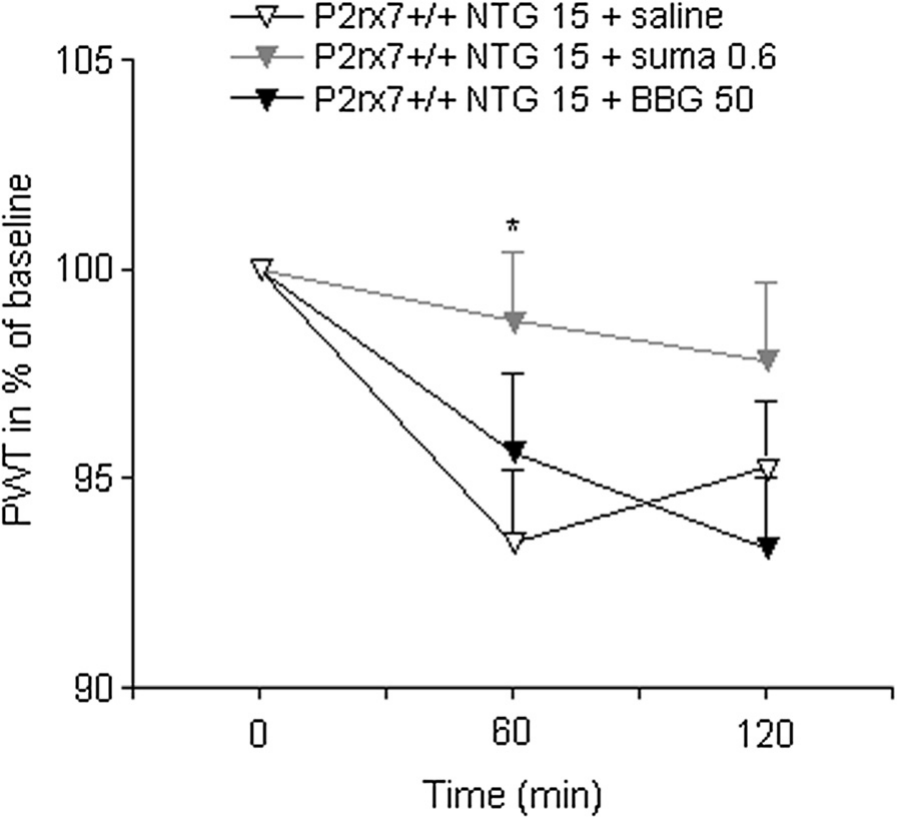
**The effect of symptomatic treatment with sumatriptan and P2X7-selective antagonist BBG.** Wild-type mice were treated with 600 μg/kg sumatriptan/50 mg/kg BBG i.p. or an identical volume of saline 5 min after NTG treatment. PWT is expressed in % of baseline. Asterisk indicate significant changes between respective PWT values of saline- and sumatriptan-treated mice (n = 10 animals/group; ANOVA + Fischer LSD post hoc test, *P < 0.05).

For the validation of the model, the antimigraine drug sumatriptan (600 μg/kg i.p.) was chosen as it is known to reverse NTG-induced thermal hypersensitivity in mice [14]. In P2X7+/+ mice, treated with sumatriptan 5 min after NTG, PWT was higher than in mice treated with an identical volume of saline (at 1 h: 98.74 ± 1.66%, n = 10; 93.43 ± 1.80%, n = 10, p = 0.0375 in NTG + sumatriptan and NTG + saline treated mice, respectively, Figure 2).

In P2X7-/- mice, the baseline PWT values were not significantly different from P2X7+/+ mice (45.47 ± 0.23°C, n = 20; p = 0.1015; Student’s *t*-test). Likewise, the NTG-induced decrease in PWT was not significantly different in wild-type and knockout mice (ANOVA genotype × treatment effect F_1,41_ = 1.00, p = 0.3243; Figure 1).

### P2X7R antagonist prevents NTG-induced thermal hypersensitivity

**Figure 3:**
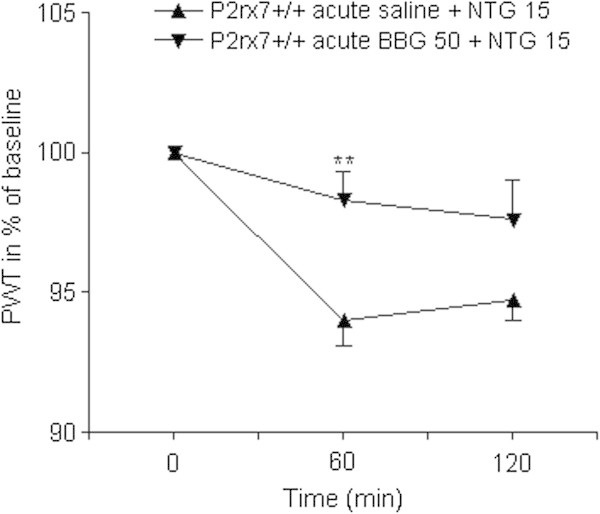
**Acute prophylactic BBG treatment alleviates NTG-induced thermal hypersensitivity in wild-type mice.** Mice were treated with 50 mg/kg BBG i.p. or an identical volume of saline 30 min before NTG treatment. PWT is expressed in % of baseline. Asterisks indicate statistical significant difference in respective PWT values from the saline-treated animals (n = 11-12 mice/group; Student’s *t*-test, **P < 0.01).

**Figure 4:**
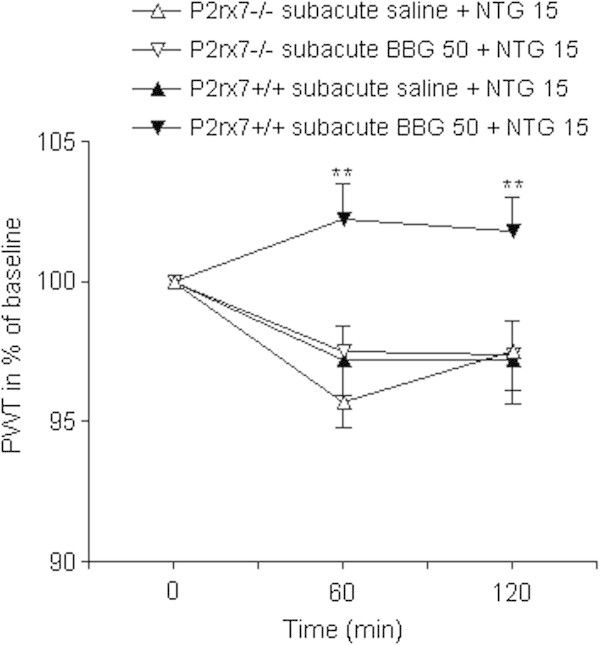
**Subacute treatment with BBG reverses NTG-induced thermal hypersensitivity in wild-type mice, but not in P2X7 knockout mice.** Mice were treated for 5 consecutive days with daily doses BBG (50 mg/kg i.p.) or saline and on the fifth day NTG was given 30 min after BBG or saline and then submitted to the ITHT. PWT is expressed in % of baseline. Asterisks indicate significant changes between respective PWT values of saline- and BBG-treated mice (n = 9-16 animals/group; ANOVA + Fischer LSD post hoc test, **P < 0.01).

Next, we asked whether systemic administration of a specific P2X7 antagonist, BBG, can alleviate NTG-induced thermal allodynia in mice. When administered 5 min after the NTG injection, BBG had no effect on thermal hypersensitivity in wild-type mice (Figure 2). However, when BBG was given as a prophylactic agent, 30 min before administration of NTG, it was already effective upon single application (Figure 3), and it completely prevented the effect of NTG after 5-days treatment in wild-type mice. In contrast, an identical BBG administration was ineffective in NTG-treated P2X7 knockout mice (ANOVA genotype × treatment effect F_1,45_ = 6.36, p = 0.0153; Figure 4). The 5-day treatment with BBG did not change the baseline thermal sensitivity of either genotype (P2X7+/+: 44.38 ± 0.27°C, n = 16; p = 0.9929, P2X7-/-: 44.86 ± 0.39°C, n = 17; p = 0.167 vs. baseline PWT, Student’s *t*-test).

### NTG induced c-fos expression in trigeminal nucleus and spinal cord

**Figure 5:**
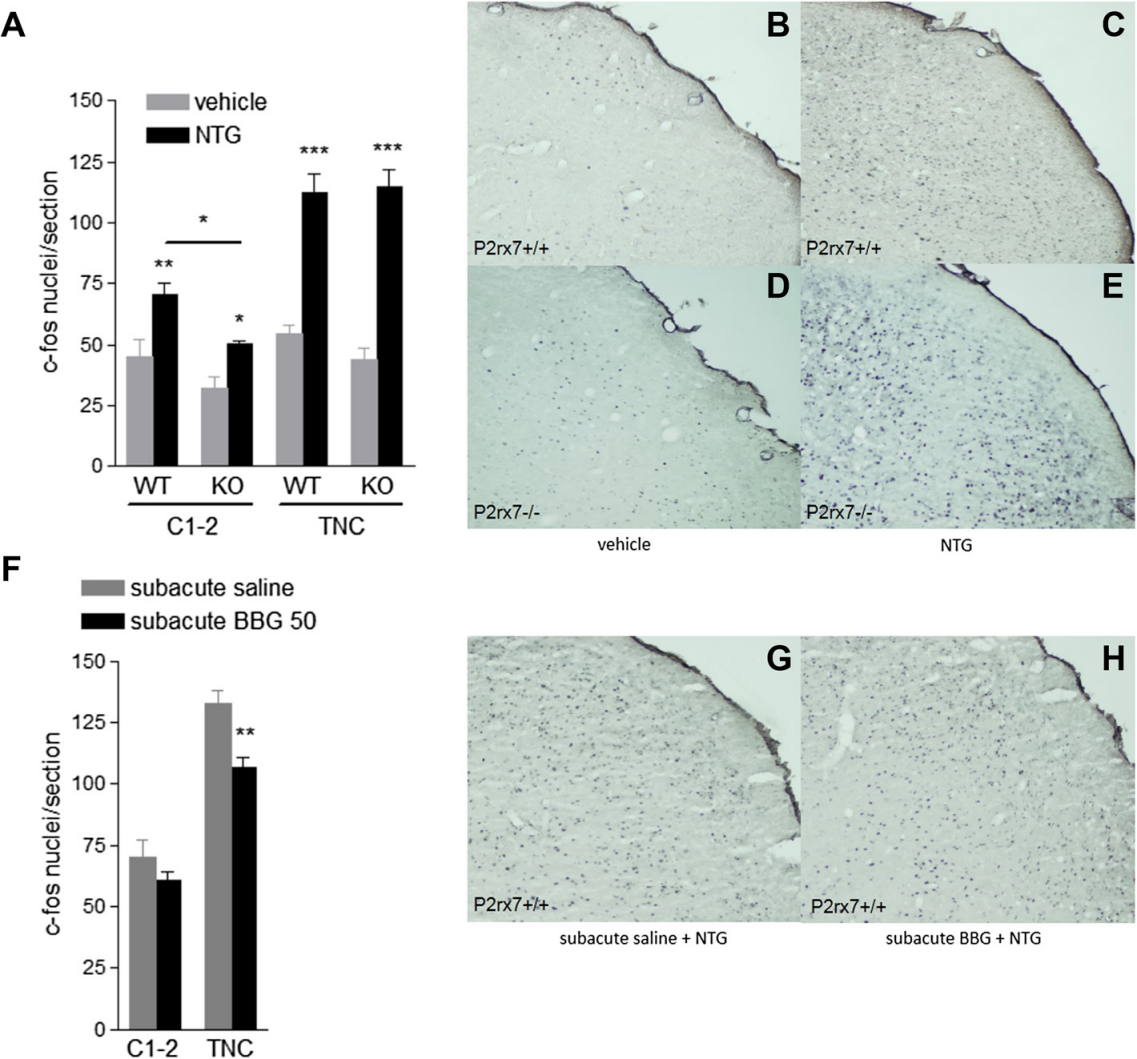
**NTG-induced fos expression in wild-type and P2X7 knockout mice. A.** Quantification of fos-immuno-reactive nuclei in upper cervical spinal cord (C1-2) and TNC 2 h after 15 mg/kg NTG or vehicle. We counted 10 hemi-sections from each hemisphere for upper cervical spinal cord, and 30 hemi-sections from each hemisphere that included TNC from both sides (n = 5-6 animal/group; Kruskal-Wallis nonparametric ANOVA followed by Fischer LSD post hoc test, *P < 0.05, **P < 0.01, ***P < 0.001). **F.** The subacute BBG treatment (50 mg/kg i.p.) decreased the number of c-fos immuno-reactive cells in TNC, but not in C1-2 in wild-type mice (n = 6-8 animal/group; Student *t* test **P < 0.01). **B,C,D,E,G,H** Representative examples of c-fos immuno-reactivity in the TNC 2 h after i.p. administration of vehicle or 15 mg/kg NTG in P2X7+/+ **(B,C)** and P2X7-/- mice **(D,E)**, and after subacute BBG (50 mg/kg i.p.) or saline + 15 mg/kg NTG treatment in P2X7 +/+ mice **(G,H)**, shown here with a 10× objective.

As an index of activation of the nociceptive fibres implicated in migraine, changes in the average number of c-fos-immuno-reactive nuclei in the upper cervical dorsal horn and TNC 2 h after 15 mg/kg NTG injection were quantified. In line with previous findings [14,15], NTG profoundly increased c-fos expression in both areas, when compared to vehicle-treated animals (Figure 5). In knockout mice, NTG caused the same elevation in c-fos expression in TNC, compared with vehicle, whereas the c-fos level was slightly lower in the spinal cord (Figure 5A). Two-way ANOVA of the c-fos data in the C1-2 and TNC indicated a significant effect of NTG treatment, without an interaction effect of genotype with NTG (C1-2: F_1,19_ = 13.92, p = 0.0014; TNC: F_1,19_ = 48.14, p < 0.0001). Due to unequal variance, a Kruskal-Wallis nonparametric test was used for comparison of groups. It indicated that NTG significantly increased c-fos levels in the C1-2 of wild-type (WT: p = 0.0374; KO: p = 0.0679) and TNC of wild-type and knockout mice, respectively (WT: p = 0.0039; KO: p = 0.0062). Further, post hoc testing by Fischer LSD test revealed that NTG significantly increased fos level in the C1-2 and TNC of wild-type and knockout mice (Figure 5A).

5-day treatment with the P2X7 antagonist BBG (50 mg/kg i.p.) elicited a reduction in the level of c-fos after NTG treatment in TNC in wild-type mice, which was significant, when compared to an identical saline treatment (Figure 5F, G and H, 106.63 ± 3.99, n = 8; 132.68, n = 6; p = 0.0022, in BBG + NTG and saline + NTG treated mice, Student *t* test, respectively).

## Discussion

The principal new finding of the present study is that antagonism of P2X7 by treatment with the specific P2X7 antagonist BBG leads to the alleviation of NTG-induced thermal hypersensitivity in mice. Moreover, as BBG treatment was ineffective in mice lacking P2X7, it is reasonable to assume that its effect is mediated by P2X7. The NTG-evoked c-fos expression in the TNC was also attenuated after subacute BBG treatment, which implicates a role for the TNC in mediating the effect of BBG on NTG-induced thermal hypersensitivity. All these findings implicate the therapeutic potential of P2X7 blockade in migraine.

We have used the NTG-induced migraine model described recently by Bates et al. [14] and reproduced findings showing that i.p. injection of NTG elicits thermal hypersensitivity in a time-dependent manner. We have also replicated the finding that NTG-induced thermal hypersensitivity is attenuated by the antimigraine drug, sumatriptan, and is followed by the expression of c-fos in the trigeminal nucleus of the brainstem and upper spinal cord [14]. Although the dose of NTG necessary to induce a significant drop in PWT (15 mg/kg), was somewhat higher than used by Bates et al. [14], this may be explained by differences in the source of NTG, or by the variation in the tests used to evaluate thermal nociception (Hargreaves test vs. ITHT). The interspecies variations in NTG sensitivity are well known and may be due to differences in the efficiency of hepatic bioactivation of NTG into the pharmacologically active NO [40]. This might also account for slight differences between NTG responsiveness observed in different rodent species [14-16].

Following the study of Almási et al. [41], we have used the ITHT to measure thermal nociceptive threshold, in our experiments instead of the widely used latency measurements. As reported in this study, ITHT is a more accurate assay of thermal hyperalgesia although the changes in response to traditional analgesic drugs, such as diclofenac and paracetamol, are not more than 2.0-2.5°C in this test. Accordingly, similar changes were measured, in response to the antimigraine drug sumatriptan, indicating that the assay system reflects changes specific to antimigraine therapy.

Interestingly, no differences in NTG-induced thermal hypersensitivity in P2X7-/- mice were detected, when compared to their wild-type counterparts. The most likely explanation for this negative finding is the potential developmental upregulation of non-P2X7 P2X receptors in P2X7-/- mice, such as P2X3 or P2X4. In fact we have previously shown the upregulation of P2X4 mRNA in mice deficient of P2X7 [42].

When applied prophylactically, however, both acute and subacute BBG treatment was effective in the alleviation of NTG-induced thermal hypersensitivity in the P2X7+/+mice. BBG is known to permeate the blood brain barrier, and is thought to be specific to P2X7 in the applied dose. Although in vitro experiments revealed that BBG can inhibit Na^+^ channels in micromolar concentrations [43], in in vivo studies using a similar dose (45.5 mg/kg), BBG has not reached higher concentrations in the brain than 200 nM [44], which is selective for P2X7 [45]. The finding that its effect was completely lost in P2X7-/- mice also refutes the possibility of other target for BBG than P2X7 in these experiments.

Although further experiments are necessary to clarify this issue, there are several potential mechanisms, whereby endogenous P2X7 could participate in the sensitization of the trigeminovascular system. ATP is a well-known danger-signal, which is released in response to cell injury, inflammation, mechanical and metabolic distress, and is *per se* an algogenic substance [46,47]. P2X7 is a ligand-gated cation channel with high Ca^2+^ permeability, which participates in pain transmission in various ways. In the dorsal root ganglia P2X7 is expressed on satellite glia and potentiate P2X3 receptor mediated signalling by the release of pro-inflammatory cytokine TNF-α [48]. An analogous mechanism might also play a part in migraine, as satellite glia of the trigeminal ganglion express P2X7 [49] and P2X3 receptors participate in craniofacial pain by interacting with NGF, substance P and CGRP [20,22]. P2X3 receptors also display an enhanced activity in a genetic animal model of migraine [50]. Alternatively, P2X7 antagonists may act more centrally, at the level of the upper cervical spinal cord or trigeminal nucleus. P2X7 modulates the afferent nociceptive information processing within the dorsal horn of the spinal cord [35] and its activation participates in the central sensitization underlying hindpaw hyperalgesia [34]. Because moderate-high density of P2X7 receptor binding sites were found in the grey matter of the trigeminal nucleus [28] the detected alleviation of NTG induced c-fos expression in response to BBG indicates that the TNC is a potential target area for the action of P2X7 receptor antagonists. The activation of P2X7 releases excitatory amino acids in this area [27], the blockade of which might underlie the action of BBG.

Furthermore, P2X7 is also expressed in other areas of the brain [28]; therefore, a supraspinal action through diencephalic, brainstem or cortical regions cannot be excluded [51]. Finally, P2X7 is expressed on circulating and locally recruited immune cells and the best characterized action of P2X7 activation is its role in the posttranslational processing of pro-inflammatory cytokines, IL-1β and TNF-α [30,37,52], which are also known algogenic substances.

## Conclusion

The data present here indicates that inhibition of P2X7 might be a potential target for the prophylaxis of migraine. Moreover, because BBG is a closed structural analogue of a US Food and Drug Administration (FDA)–approved non-toxic food dye [53,54], our data argues for its evaluation in a human NTG-induced migraine model.

## Abbreviations

ANOVA: Analysis of variance; ATP: Adenosine triphosphate; BBG: Brilliant Blue G; C1-2: Upper cervical spinal cord; CGRP: Calcitonin gene related peptide; CNS: Central nervous system; DNA: Deoxyribonucleic acid; FDA: Food and Drug Administration; ITHT: Increasing temperature hot plate test; IHC: Immunohistochemistry; IL-1β: Interleukin-1 beta; NGF: Nerve growth factor; NO: Nitric oxide; NTG: Nitroglycerin; P2X7: P2X7 receptor; PCR: Polymerase chain reaction; PWT: Paw withdrawal threshold; SEM: Standard error of the mean; TNC: Trigeminal nucleus caudalis; TNF-α: Tumor necrosis factor-α.

## Competing interests

The authors declare that they have no competing interests.

## Authors’ contributions

FG carried out all experiments, analyzed data, performed statistical analyses and drafted the manuscript. BS designed and supervised the study, and finalized the paper. Both authors read and approved the final manuscript.
